# TIMo—A Dataset for Indoor Building Monitoring with a Time-of-Flight Camera

**DOI:** 10.3390/s22113992

**Published:** 2022-05-25

**Authors:** Pascal Schneider, Yuriy Anisimov, Raisul Islam, Bruno Mirbach, Jason Rambach, Didier Stricker, Frédéric Grandidier

**Affiliations:** 1 DFKI—German Research Center for Artificial Intelligence, 67663 Kaiserslautern, Germany; yuriy.anisimov@dfki.de (Y.A.); raisulzaeem@gmail.com (R.I.); bruno.mirbach@dfki.de (B.M.); didier.stricker@dfki.de (D.S.); 2 IEE S.A., L-7795 Bissen, Luxembourg; frederic.grandidier@iee.lu

**Keywords:** time-of-flight, depth imaging, person detection, anomaly detection, dataset, machine learning, deep learning, neural networks

## Abstract

We present TIMo (*T*ime-of-flight *I*ndoor *Mo*nitoring), a dataset for video-based monitoring of indoor spaces captured using a time-of-flight (ToF) camera. The resulting depth videos feature people performing a set of different predefined actions, for which we provide detailed annotations. Person detection for people counting and anomaly detection are the two targeted applications. Most existing surveillance video datasets provide either grayscale or RGB videos. Depth information, on the other hand, is still a rarity in this class of datasets in spite of being popular and much more common in other research fields within computer vision. Our dataset addresses this gap in the landscape of surveillance video datasets. The recordings took place at two different locations with the ToF camera set up either in a top-down or a tilted perspective on the scene. Moreover, we provide experimental evaluation results from baseline algorithms.

## 1. Introduction

Traditionally, surveillance cameras are RGB or IR cameras. For the realization of robust automatic building management functions, time-of-flight depth cameras offer, however, some unique benefits. First of all they are more robust to illumination and color variations, and allow natural geometrical background removal. The depth information they provide allows to detect, classify and localize persons and objects precisely in 3D space. Moreover people are much less likely to be identifiable in depth data compared to RGB. Thus, monitoring places and at the same time preserving peoples’ privacy can be reconciled to a much better degree. For these reasons, the first building management systems based on time-of-flight technology have been around for about ten years [1], which realizes a few basic building management functions such as people counting or single access control. Future time-of-flight sensors with higher sensitivity and resolution (see [2,3] with references therein) in combination with novel deep-learning algorithms promise to both enhance the performance of existing building management systems and realize novel functions as the behaviour analysis of persons and detection of anomalous situations [4,5].

The affordability of consumer depth cameras has made them a popular and widely used sensing device for the computer vision research community. Examples of frequently used depth cameras in this context are the Microsoft Kinect [6], Intel RealSense [7], or the Asus Xtion [8]. The fact that depth cameras provide 3D geometric information of the scene is beneficial for various various applications, such as action recognition [9] or gesture classification [10]. With our introduced dataset TIMo, we want to foster research towards robust and high performing person detection and novel smart building functions such as the detection of anomalies.

**Figure 1:**
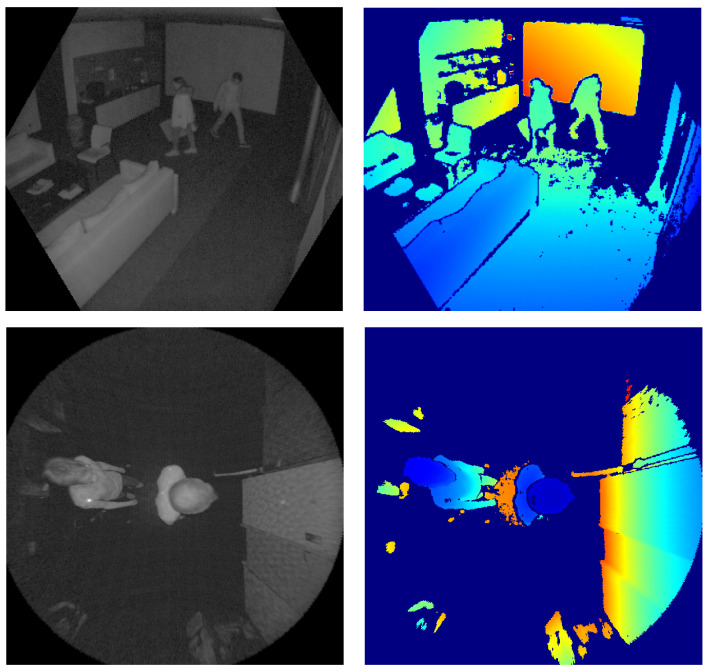
Example frames from our dataset. (**Top**): a scene from tilted view, (**Bottom**): a scene from top-down view. (**Left**): IR image, (**Right**): Depth image with depth encoded as color.

An advantage of our dataset over existing ones is the choice of more modern hardware. Our recordings were captured using a Microsoft Azure Kinect camera, which features higher image resolution, higher field-of-view and lower distance error compared to the Microsoft Kinect V2 [2,11]. In addition to the depth images, we also provide the corresponding infrared images, since they are a byproduct of the IR-based ToF depth measurement principle and comparisons between the depth and IR modality are thus also possible with the dataset. Examples of both IR and depth images are shown in Figure 1.

Some anomaly datasets additionally suffer from a very limited number of anomalous events. This is sometimes due to the fact that they use actual surveillance footage where anomalies tend to be rare. While there are advantages to using data from real-world anomalies, it can severely limit the amount of data available for testing anomaly detection algorithms. Our dataset features a large number of anomalies that facilitates testing at scale.

The rise of deep neural networks as the primary approach for solving computer vision problems leads to a demand for very large datasets that support training and testing complex models. Our dataset consists of about 1500 recordings with 44 different subjects and in total sums up to more than 612,000 individual video frames, which makes it suitable for the development of data-hungry approaches. Additionally, 243 sequences with 22,700 labeled frames with 3D bounding boxes and segmentation mask are provided for person detection with counting purposes. It is moreover relatively easy to generate high quality synthetic data for depth videos in case the real data is not sufficient, since computation of depth is a customary step of computer graphics pipelines anyway. The TiCAM dataset is an example of how synthetic depth data can complement real data [12].

The rest of the paper is structured as follows: Section 2 puts our dataset into context with respect to existing ones. Section 3 then details the content and process of acquisition of our recordings and the accompanying annotations. We provide results of baseline algorithms for the target applications in Section 4 and conclude our work in Section 5.

## 2. Related Work

This section gives an overview of the landscape of existing datasets and a brief summary of related work on anomaly detection and person detection, as those are the two main application the dataset is aimed at being used for.

### 2.1. Datasets

The popularity of depth-sensing technology has led to a large number of datasets to be released featuring RGB videos with an additional depth channel, or, in short, RGB-D. While pure RGB datasets can sometimes be compiled using existing recordings (such as videos on online video platforms), RGB-D data usually has to be recorded specifically for the dataset. A review of some of these datasets is given in [13]. Table 1 shows an overview of a number of datasets related to the one we present either by providing similar data modalities, annotations or aiming at a similar estimation problem. Some provide static scans of indoor spaces, e.g., Stanford 2D-3D-Semantics [14] or ScanNet [15] and are commonly used for tasks such as semantic segmentation of point clouds. Our dataset differs from this type of datasets in that it does not focus on the reconstruction of the static geometry of the indoor space but instead on capturing human action performed within the scene. In this regard, it is more similar to datasets used for action recognition such as UTD-MHAD [16] or NTU RGB+D [17] and NTU RGB+D 120 [18]. However, these datasets were not created for the applications of anomaly detection or people counting. The camera angles and the nature of the recorded scenes make them unsuitable for these tasks. Therefore, there is a need for datasets particularly geared towards the development of algorithms for monitoring indoor spaces.

Existing datasets for anomaly detection usually provide only RGB data, e.g., the Shanghai-Tech dataset [19] or UCF-Crime [20]. Our dataset fills this existing gap by providing depth data of realistic scenarios and following camera angles as they would be common in a surveillance context.

Unlike some other datasets, such as [18], we did not aim for a large variety of backgrounds or illumination in the data. This limitation is a consequence of the time-consuming process of calibration and the risk of having correlations between the background and the content of sequences, which has the potential to compromise the learning process and results when using machine learning. In addition, background variations are less relevant in depth data. We therefore committed to record only a few scenes and put more focus on a large and well-balanced variance of subjects and actions and on providing high quality supplementary information, e.g., camera calibration parameters.

### 2.2. Anomaly Detection

A good general overview of techniques for detecting anomalies—or *outliers* depending on the terminology used—can be found in [21].

Research towards video anomaly detection (VAD) became a more popular research topic in the course of the mid- to late-2000s. A common technical base of the methods from that era are optical flow estimations and object trajectories to which, e.g., a Hidden Markov Models is the applied technique [22,23].

In the more recent past, anomaly detection research has followed the general direction of pattern recognition research and is now mostly dominated by deep learning-based methods [24,25,26]. Examples of how VAD is approached using deep learning include learning representations of normality using autoencoder-based architectures [27] or—if the problem is posed in a supervised way—e.g., contrastive learning [28].

### 2.3. Person Detection

Overall the topic of depth-based or depth-supported person detection is not widely presented in state-of-the-art publications. There are some noticeable publications, methods of which can potentially utilize and benefit from the presented dataset. The work of Tan et al. [29] describes a method for pedestrian detection based on RGB-D information with improved HHA encoding of depth maps [30]. The approach from [31] learns an upper body person template from depth images for further close-range person detection. For the task of mobile person detection, the approach of Choi et al. [32] utilizes depth images from early generation of Kinect camera, which are a subject of segmentation with further candidate classification. In a method by Xia et al. [33], depth images from Kinect are used to generate a contour based on the Canny edge detector [34]. A head template is matched to the resulting image in order to find the person’s location. For the mechanical safety applications, the approach of Zhou et al. [35] detects a person in RGB frames and then finds the associated distance to the person to prevent entering the dangerous area. Wetzel et al. [36] use a fusion of information from multiple depth cameras for person detection from different viewpoints.

**Table 1:** Comparison of related datasets to our dataset. The 3D joints refers to joints of the human body such as they are used in human pose estimation.

Dataset	Year	# Sequences (# Frames)	Data Modalities	Camera Hardware	Annotations	Environment
TIMo Anomaly Detection (ours)	2021	1588 (612 K)	IR, Depth	MS Kinect Azure	Anomaly Frames	Indoor
TIMo Person Detection (ours)	2021	243 (23.6 K)	IR, Depth	MS Kinect Azure	2D/3D Object BBox, 2D Segm. Masks	Indoor
ShanghaiTech Campus [19]	2018	437 (317 K)	RGB	RGB Camera	Anomaly Frames, Anomaly Masks	Outdoor
UTD-MHAD [16]	2015	861 (45 K)	RGB, Depth, 3D Joints, ID	MS Kinect v1	Action Classes	Indoor
NTU-RGB+D 120 [18]	2019	114 K (4 M)	RGB, Depth, 3D Joints, Inertia	MS Kinect v2	Action Classes	Indoor
UCF-Crime [20]	2018	1900 (13.8 M)	RGB	RGB Camera	Anomaly Frames	Indoor + Outdoor
TiCAM (Real) [12]	2021	533 (6.7 K/118 K)	RGB, IR, Depth	MS Kinect Azure	2D/3D Object BBox, 2D Segm. Masks Action Classes	Car Cabin
DAD [28]	2020	386 (2.1 M)	IR, Depth	CamBoard pico flexx	Anomaly Frames	Car Cabin
CUHK Avenue [37]	2013	37 (31 K)	RGB	RGB Camera	Anomaly Frames, Anomaly BBoxes	Outdoor
UCSD Ped 1 + 2 [38]	2010	70 + 28 (14 K + 4.6 K)	Grayscale	Grayscale Camera	Anomaly Frames, partly Anomaly Masks	Outdoor
Subway Exit + Entrance [23]	2008	1 + 1 (137 K + 72 K)	Grayscale	Grayscale Camera	Anomaly Frames, rough Anomaly Locations	Subway Station
IITB-Corridor [39]	2020	368 (484 K)	RGB	RGB Camera	Anomaly Frames	Outdoor (Corridor)

## 3. TIMo Dataset

Here we describe the content of the dataset as well as the process of recording. Section 3.1 and Section 3.3 cover the data modalities, the choice of hardware and scene and how recordings were carried out. Section 3.4, Section 3.5 and Section 3.6 give more details about the content and annotations we provide in the dataset. Section 3.7 gives a quantitative overview of the dataset in the form of statistics.

### 3.1. Data Modalities

We provide the infrared (IR) images and the depth maps estimated by the Microsoft Azure Kinect camera. The camera in principle also features recording RGB images, which we use in some of the figures for visualization purposes. Note, however, that our focus lies on depth data and hence we do not provide RGB frames in the public dataset and there are also no annotations for the RGB modality.

### 3.2. Setup

**Figure 2:**
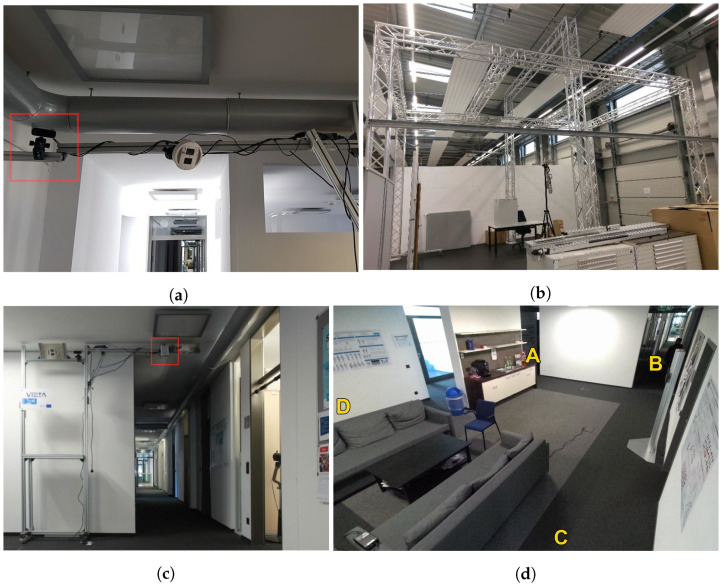
Recording setups used for capturing the dataset. The position of the Azure Kinect is marked with a red square. (**a**) Camera mounting for tilted-view in Scene 1. (**b**) Setup for top-down view in Scene 2 (camera not installed yet). (**c**) Setup for top-down view in Scene 1. (**d**) Entrances in in Scene 1. Subjects were told to use specific entrances during recording (e.g. enter at A and leave through D).

Recordings took place at two different locations, which we refer to as Scene 1 and Scene 2. Scene 1 is an open office area with a small kitchen and a seating area. For this scene the Microsoft Azure Kinect camera was installed in two different positions. There was a tilted-view mounting in which the camera was able to monitor a large portion of the room including the four entrance possibilities, which are shown in Figure 2d. The sequences were recorded in such a way that unwanted correlations between the occurrence of an anomalous event and the entrances used by the actors are avoided. In addition, the camera was mounted on a metal frame above the entrance B with a top-down view, monitoring people entering or leaving the room through a hallway.

The same top-down mounting orientation has been used in Scene 2 but with more flexibility. There, the monitored area is less confined, allowing persons to cross the scene in all directions. Moreover, the camera is mounted on a lift, allowing to vary the camera height. Figure 2a–c show the different camera mounting setups. For the two different mounting orientations, the camera configuration was different, as described below.

An important advantage of our recording setup compared to most other ones shown in Table 1 is the use of the latest generation of time-of-flight sensor technology. We can thus achieve measurements with less noise and higher resolution. For a detailed comparison of the Kinect devices, we refer to [2,11].

#### 3.2.1. Top-Down View

For top-down recordings we used the wide field-of-view (WFOV) configuration of the Azure Kinect (120 ${}^{{}^{\circ}}$× 120 ${}^{{}^{\circ}}$). The native resolution of the sensor in WFOV configuration is 1024×1024 pixels. We employed a 2×2 binning technique which reduces the resolution down to 512×512 pixels but at the same time yields a higher operating range, which is 0.25 m–2.88 m. The capturing rate is set to 30 frames per second (FPS). Top-down view data were recorded in Scene 1 and Scene 2. The camera height above ground was in Scene 2 varied between 2.25 m, 2.50 m and 2.75 m, while in Scene 1 it was fixed to 2.50 m.

#### 3.2.2. Tilted-View

All tilted-view data were recorded in Scene 1. For these recordings we used the narrow field-of-view (NFOV) configuration (75 ${}^{{}^{\circ}}$× 65 ${}^{{}^{\circ}}$). The tilt angle is roughly 41 ${}^{{}^{\circ}}$. The native resolution of the sensor in NFOV configuration is 576×640 pixels. Same as for the top-down configuration, we used a frame rate of 30 FPS and 2×2 binning, which results in a resolution of 288×320 pixels.

The maximum guaranteed depth operating range for the NFOV configuration with 2×2 binning is 5.46 m. For the recordings from the tilted-view camer, a this is not enough range to cover the complete scene. As is usual for time-of-flight cameras, the data quality also depends significantly on the remission properties of the surface in question. Nevertheless, we observed that we obtain adequate depth measurements for the relevant parts of the scene up to about 10 m in our setup.

### 3.3. Acquisition

The test cases were defined prior to recording according to a test matrix, which aims at preventing unintentionally introduced correlations in the dataset. The anomalies in the anomaly dataset also belong to pre-defined test cases. The camera was calibrated according to a world coordinate system before each recording session. For recording, the test subjects were instructed to enter and leave the scene through a specific entrance. For the tilted-view scene, there are four such entrances. In the top-down-view scenes, the test subjects cross the scene either in the *X*- or *Y*-direction of the camera coordinate system. The subjects perform a given action after entering the scene and then leave again. The instructions on how an action was to be performed were kept rather vague in order to have some degree of variance between performances of the same action type. A full list of the choreographies can be found on the dataset website https://vizta-tof.kl.dfki.de/building-data-format/ (accessed on 22 April 2022).

### 3.4. Post-Processing

Post-processing of the data and the annotations is kept to a minimum in order to allow users to choose between using the raw data or applying custom normalization themselves. Images in the person detection dataset are undistorted and remapped to the common pinhole camera model. The original rotation and translation matrices are also provided per sequence for its further conversion to the 3D world coordinate system (e.g., in the form of a point cloud). The segmentation masks and 2D bounding boxes are provided in the coordinates of the undistorted and remapped images (see Section 3.6 for more details on the annotations).

### 3.5. Data Format

The IR and depth videos are stored as individual frames in the Portable Network Graphics (PNG) format with a single 16-bit channel. The pixel values in the depth images directly correspond to the depth measurements in millimeters. A pixel value of 0 is used as a special value to indicate that there is no valid depth estimation for this pixel. Note that the example frames shown in this paper have been transformed with respect to value range and contrast for better visualization.

The video sequences and individual frames follow a common naming scheme which includes the most relevant information directly in the file name. It includes the choreography, a sequence ID, the camera height, a timestamp and a calibration ID. More details on the naming scheme can also be found on the dataset website.

### 3.6. Annotations

We provide annotations for people and objects in the form of 2D and 3D bounding boxes and segmentation masks for the person detection dataset and anomaly annotations for the anomaly dataset. For the anomaly dataset, both tilted view data recorded in *Scene 1* and top-down data recorded in *Scene 2* were annotated. For the person detection dataset, data from both scenes recorded in the top-down configuration were annotated. The annotations were done manually using a software tool that was developed specifically for the purpose of annotating 3D data [40].

#### 3.6.1. Anomaly Annotations

Anomaly annotations are provided as pairs of frame indices which indicate when the anomalous event within the given sequence starts and ends. Note that the frames at START_FRAME_IDX and END_FRAME_IDX both also belong to the anomalous event, thus making it END_FRAME_IDX−START_FRAME_IDX + 1 frames long. A sequence contains either exactly one anomalous event or none at all. The anomalies can involve a single person (e.g., collapsing) or multiple people (e.g., arguing). We thus consider the whole scene depicted by a certain video frame as either normal or anomalous depending on the behaviour it features. Our definition of anomalous events and corresponding choreographies in the recorded videos was inspired by related work, but is of course context dependent. We therefore also provide the annotation of the choreography shown in a video. By labelling each choreography either as normal or anomalous (or omitting it), the ground truth of the dataset can be adjusted to the context and use case considered.

**Figure 3:**
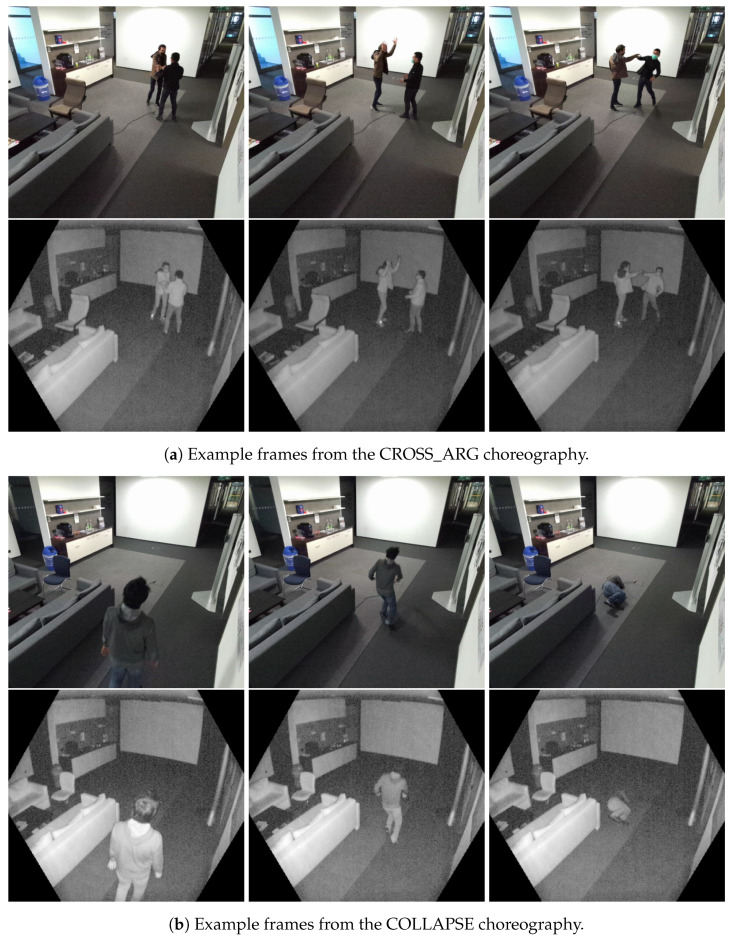
Example frames of anomalies. Top row: RGB, bottom row: Infrared. (Please note that the RGB data modality is only used for visualization here and not provided in the dataset).

Examples of anomalies include left-behind objects, people arguing or throwing objects. Figure 3 illustrates two instances of anomalous events within the dataset.

All choreographies that are not labeled as anomalous are consequently considered to be normal. This includes activities such as getting coffee from the kitchen, talking with one another or simply walking.

#### 3.6.2. Person and Object Annotations

For person detection, we provide annotations for people and objects in the following form:

- Two-dimensional (2D) segmentation masks per frame, saved as 8-bit PNG images. Pixel values correspond to class and instance IDs respectively;
- Two dimensional (2D) bounding boxes per annotation, described as pixel coordinates of rectangular around the annotated object. This data is presented in corresponding CSV in a form of $\left[x_{1},y_{1},x_{2},y_{2}\right]$, where $\left(x_{1},y_{1}\right)$ and $\left(x_{2},y_{2}\right)$ are the coordinates of upper left and lower right corner of the bounding box;
- Three-dimensional (3D) bounding boxes per annotation, presented in corresponding CSV as a box center (cx,cy,cz) and its dimensions (dx,dy,dz) in the world coordinate system.

Objects are annotated as a separated class only if they are not held by a person at the specific frame. The 3D bounding boxes are generated automatically using the segmentation masks, since the calibration of the camera allows a direct mapping between the 2D image space and the 3D point clouds implicitly given in the form of the depth maps. We also used interpolation between manual annotations to speed up the process in situations where this could be done without impairing the quality of the annotations. All annotations were additionally validated by a person different from the one who created the annotation.

**Figure 4:**
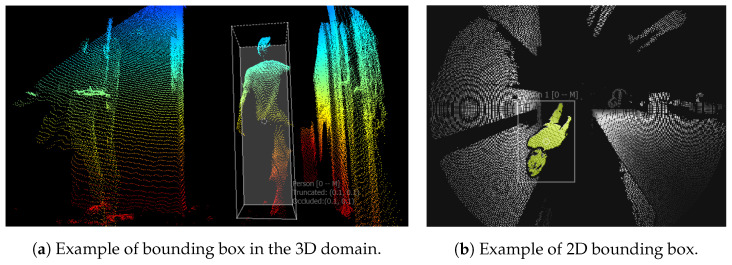
Visualization of annotations for the person detection/people counting dataset, generated by [40].

Examples of these annotations are illustrated in Figure 4.

### 3.7. Data Statistics

Table 2 and Table 3 show the splits of datasets in training and testing data. Both the training and testing set have been further split according to the complexity of the scenes. Scene 2 data were recorded with a camera mounted at different heights, and in this scene the variety of movements is greater than in Scene 1, which explains the choice of these sequences for training. In contrast, data from Scene 1 were captured with the camera mounted at 2.50 m and were suggested to be used for testing. Complex top-down sequences were captured at Scene 2 and split to training and testing sets based on the captured person and his activity.

The data splits are designed for usage with unsupervised learning techniques. Therefore, the training set only consists of normal sequences. The test set mostly consists of sequences that contain anomalies, but also contains some normal sequences as well. This aims at facilitating the evaluation of the false positive rate. Because of the two different camera configurations used in the recordings in Scene 1, both training and test set are split accordingly.

## 4. Baseline Results

We provide baseline results for both anomaly detection and people counting based on recent methods in the respective research area in the following. Since the main contribution of the paper lies in the presentation of the a new dataset, the baseline algorithms are derived from existing methods for RGB data.

### 4.1. Anomaly Detection Baseline

For anomaly detection, we employ an approach based on convolutional autoencoders (CAE), which is one of the most commonly used methods in this context. The working principle is learning a latent representation of normality by having the CAE learn to reconstruct frames from the training set and computing loss as the mean squared error (MSE) between input and reconstruction. When faced with frames from anomalous events, the reconstruction by the network is expected to cause higher MSE. The loss is thus interpreted as the anomaly score. Since the training set consists only of normal samples, the resulting anomaly detection approach is unsupervised. This approach was described by Hasan et al. [27]. Our implementation uses a network with an encoder stage of three convolutional layers with a kernel size of 5×5 and a 2×2 pooling layer after each convolutional layer and a symmetric decoder stage. The filter size is reduced from initially 32 down to 8 in the latent space and then increased to 64 again before the last convolutional layer performs the frame reconstruction.

We additionally evaluated the results from a network based on the concept of a Convolutional LSTM to the data [41]. It was previously already successfully used in the context of VAD for RGB data [19]. Our specific model of the network consists of 6 ConvLSTM cells with a number of 8 hidden dimensions in each cell.

The performance is measured as frame-level area under the ROC curve (AUROC). The results for both parts of the dataset—tilted view and top-down view—are reported in Table 4. These results indicate that the dataset is challenging, but still in the realm of what is possible to be approached with recent methods.

### 4.2. Person Detection Baseline

The person detection dataset was evaluated with two instance segmentation network architectures: Mask R-CNN [42] and YOLACT [43]. The original implementation was modified to accept the original depth data instead of RGB. The evaluation of algorithms is based on the mean average precision (mAP) metric and presented in Table 5. Results of this evaluation show that the object detection and instance segmentation algorithms can be trained on the proposed dataset with an acceptable level of accuracy. However, due to the high requirements of most applications in the context of this task to reliably achieve very high detection rates, the dataset appears to be adequately challenging for future research and further improvements.

## 5. Conclusions

We presented an extensive dataset of video sequences for monitoring indoor scenes consisting of IR and depth videos captured by a time-of-flight camera of the latest generation. It consists of about 1600 sequences with a total of roughly 600,000 frames for the anomaly detection use case and about 240 sequences and 24,000 frames for the person detection and people counting use case. We described the data and the associated annotations as well as the recording setup and process. The dataset aims at facilitating the development of depth-based algorithms for monitoring indoor spaces in order to allow such functionality to be implemented in a more privacy-preserving way.

## Figures and Tables

**Table 2:** Data statistics of the anomaly dataset’s train and test split. The train split does not contain anomalies since the split was made for usage with unsupervised methods. Note that some choreographies are used in both the tilted view as well as the top-down view, so the total number of unique choreographies is less than the sum from the configurations.

TIMo Anomaly Dataset–Train Split								
**Configuration**	**# Sequences**			**# Frames**			**Unique Choreographies**	
	**Normal**	**Anomalous**	**Total**	**Normal**	**Anomalous**	**Total**	**Normal**	**Anomalous**
Tilted View	285	0	285	185,620	0	185,620	31	0
Top-down View	624	0	624	180,359	0	180,359	19	0
Total	909	0	909	365,979	0	365,979	36	0
**TIMo Anomaly Dataset–Test Split**								
**Configuration**	**# Sequences**			**# Frames**			**Unique Choreographies**	
	**Normal**	**Anomalous**	**Total**	**Normal**	**Anomalous**	**Total**	**Normal**	**Anomalous**
Tilted View	31	151	182	66,508	25,617	92,125	29	20
Top-down View	79	418	497	104,165	49,528	153,693	18	12
Total	110	569	679	170,673	75,145	245,818	34	22

**Table 3:** Data statistics of the TIMo person detection dataset.

TIMo Person Detection Dataset			
**Data Type**	**Sequences**	**Frames**	**Annotations**
Training	125	6415	8501
Complex Training	34	7675	8186
Total	159	14,090	16,687
Testing	72	5089	6129
Complex Testing	12	3533	4971
Total	84	8622	11,000

**Table 4:** Results of our anomaly detection baseline algorithm measured as the relative area under the ROC curve (AUROC). The tilted view data was recorded at Scene 1 and the top-down view data at Scene 2.

Anomaly Detection Dataset		
**Dataset Part**	**CAE**	**ConvLSTM**
Tilted View	66.4%	62.8%
Top-down View	56.4%	62.2%

**Table 5:** Results of person detection on Mask R-CNN [42] and YOLACT [43].

Person Detection Dataset		
**Algorithm**	**mAP Box**	**mAP Mask**
Mask R-CNN	92.9 %	92.8 %
YOLACT	88.6 %	93.0 %

## Data Availability

The dataset is publicly available at https://vizta-tof.kl.dfki.de/timo-dataset-overview/ (accessed on 22 April 2022). In order to download the data, a free account has to be registered on the website first.
